# Statins during Anticoagulation for Emergency Life-Threatening Venous Thromboembolism: A Review

**DOI:** 10.3390/medicina60081240

**Published:** 2024-07-30

**Authors:** Carmine Siniscalchi, Egidio Imbalzano, Tiziana Meschi, Andrea Ticinesi, Beatrice Prati, Manuela Basaglia, Giuseppe Camporese, Alessandro Perrella, Andreev Viorica, Elisa Eletto, Vincenzo Russo, Paolo Simioni

**Affiliations:** 1 Department of Medicine and Surgery, University of Parma, Via Antonio Gramsci 14, 43126 Parma, Italy; tiziana.meschi@unipr.it (T.M.); andrea.ticinesi@unipr.it (A.T.); bprati@ao.pr.it (B.P.); mbasaglia80@gmail.com (M.B.); vandreev@ao.pr.it (A.V.); eeletto@ao.pr.it (E.E.); 2 Parma University Hospital-Azienda Ospedaliero-Universitaria di Parma, Via Antonio Gramsci 14, 43126 Parma, Italy; 3 Department of Clinical and Experimental Medicine, University of Messina, 98122 Messina, Italy; eimbalzano@unime.it; 4 Department of Medicine-DIMED, Clinica Medica 1, Padua University Hospital, 35128 Padua, Italy; giuseppe.camporese@aopd.veneto.it (G.C.); paolo.simioni@unipd.it (P.S.); 5 Infectious Disease Division, PO Cotugno, 80131 Naples, Italy; alessandro.perrella@ospedalideicolli.it; 6 Department of Cardiology, Vanvitelli University of Naples, 80138 Naples, Italy; vincenzo.russo@unicampania.it

**Keywords:** venous thromboembolism, deep vein thrombosis, pulmonary embolism, statin, blood clotting

## Abstract

Venous thromboembolism (VTE) is the leading cause of morbidity and death worldwide, after cancer and cardiovascular diseases. VTE is defined to include pulmonary embolism (PE) and/or deep vein thrombosis (DVT). Approximately 25% of PE patients experience sudden death as an initial symptom of VTE, and between 10% and 30% of patients die within the first month after diagnosis. Currently, the only drugs approved for the treatment of both acute and chronic VTE are vitamin K antagonists (VKAs) and direct oral anticoagulants (DOACs). However, their effectiveness is limited due to their associated risk of bleeding. Ideally, therapy should be able to treat VTE and limit the risk of VTE recurrence without increasing the risk of bleeding. Several studies have shown that the use of statins during anticoagulation for VTE reduces the risk of death and VTE recurrence. However, to date, there are conflicting data on the impact of statins during anticoagulation for VTE. A biological protective function of statins during anticoagulation has also been reported. Statins affect D-dimer levels; tissue factor (TF) gene expression; and VIII, VII, and Von Willebrand clotting factors—the major clotting factors they are able to affect. However, the usefulness of statins for the treatment and prevention of VTE is currently under debate, and they should not be substituted for guideline-recommended VTE prophylaxis or anticoagulation treatment. In this review of the literature, we illustrate the advances on this topic, including data on the role of statins in primary VTE prevention and secondary VTE prevention, related biological mechanisms, the risk of bleeding during their use, and their ability to reduce the risk of death.

## 1. Introduction

Venous thromboembolism (VTE) is the leading cause of morbidity and death worldwide, after cancer and cardiovascular diseases [1], and it exacts high healthcare costs [2]. Every year, more than 900,000 individuals worldwide (1 to 2 in 1000) are affected by VTE [2]. VTE is a global health problem with a high prevalence and high mortality. Approximately 25% of patients with PE (pulmonary embolism) experience sudden death as the initial symptom, and 10–30% of patients die within the first month after diagnosis. Currently, the only drugs approved for the treatment of both acute and chronic VTE are heparin, low-molecular weight heparin (LMWH), vitamin K antagonists (VKAs), and direct oral anticoagulants (DOACs) [3,4]. The main limits of these classes of drugs are their related risk of bleeding, approximately double that of placebo [3,4]. However, in patients with unprovoked VTE, the risk of recurrence increases to 10% each year when anticoagulant therapy is stopped [5], while the recurrence rate during anticoagulant therapy is 3% in the first 3–6 months [6,7]. Therefore, is questionable whether there are drugs that can reduce the risk of VTE recurrence without increasing the risk of significant bleeding and death. Ideally, therapy should be able to treat VTE and limit the risk of VTE recurrence without increasing the risk of bleeding. Several studies have shown that the use of statins during anticoagulation for VTE reduces the risk of death and VTE recurrence. Statins, by downregulation of hydroxymethylglutaryl coenzyme A reductase, are capable of inhibiting cholesterol biosynthesis and thus lower blood cholesterol levels [8]. Patients with or at risk of cardiovascular disease frequently use statins for they protective role against myocardial infarction, stroke, and cardiovascular death [9]. However, statins have several “pleiotropic” effects that are independent of their capacity to lower blood cholesterol levels [10,11]. In this review of the literature, we illustrate the advances on this topic, including data on the role of statins in primary VTE prevention and secondary VTE prevention, the related biological mechanisms, the risk of bleeding during their use, and their ability to reduce the risk of death. However, we emphasize that the usefulness of statins for the treatment and prevention of VTE is currently under debate and that they should not be substituted for guideline-recommended VTE prophylaxis or anticoagulation treatment. The structure of the review of the literature is as follows: first, we analyze the role of statins in primary and secondary prevention of VTE; then, we consider the risk of bleeding related to the use of statins during anticoagulation and the molecular mechanisms involved in the capacity of statins to influence clotting factors.

## 2. Statins and Venous Thromboembolism

### 2.1. Statin Use and Their Role in Primary VTE Prevention

A recent meta-analysis suggested that the use of statins is associated with a lower risk of incident VTE (RR: 0.78, 95 percent CI [confidence interval]: 0.72–0.85) [12]. All included studies, however, showed heterogeneity (I = 81%), and observational studies showed a lower risk of VTE (RR: 0.78, 95 percent CI: 0.72–0.85), which was not confirmed in randomized clinical trials (RCTs) included in the same meta-analysis (RR: 0.88, 95 percent CI: 0.36–2.15). Statin use was not linked to a lower risk of incident VTE in six observational studies that used propensity score methods for statistical analysis (RR: 0.92, 95 percent CI: 0.84–1.01). However, in 23 studies that used multivariable adjusted regression models, the populations that received statin treatment showed a 25% lower risk of VTE (RR: 0.75, 95 percent CI: 0.68–0.82). Several studies have demonstrated how statins can lower the risk of VTE events occurring for the first time. It has also been shown that the use of statins may play a part in the pharmacological prevention of initial VTE. About 18,000 patients with elevated hs (high sensibility) C-reactive protein participated in the randomized, placebo-controlled JUPITER study, which revealed that rosuvastatin users had a 43 percent lower chance of experiencing a first episode of VTE (OR: 0.57, CI 95 percent: 0.37–0.86) [13]. These results were supported by four cohort studies and four case–control studies, which indicated that compared to non-users, statin users had a lower risk of deep vein thrombosis (odds ratio: 0.53; 95 percent CI: 0.22, 1.29) and VTE (odds ratio: 0.67; 95 percent CI: 0.53, 0.84) [14,15]. In primary VTE prevention, the role of statins is strengthened by Mendelian randomization (MR). An MR study that made use of gene variants coding for less efficient hydroxymethyl-glutaryl coenzyme A (HMG-CoA) reductase, which were used as a proxy for statin treatment, found a small but significant negative association between genetic polymorphisms mimicking the effect of statins and the incidence of VTE, PE, and DVT.

### 2.2. Statin Use and Their Role in Secondary VTE Prevention

Several researchers have suggested that statin use may also reduce the risk of VTE recurrence [16,17,18,19,20]. A previous pool analysis conducted by Kunutsor et al. [19,21] found that statin users had a 26% lower risk of VTE recurrence compared to non-users. In a large study, statin users had a significantly lower risk of VTE recurrence compared to non-users (adjusted hazard ratio [HR]: 0.74; 95% CI: 0.68–0.80 and 0.72; 95% CI: 0.59–0.88, respectively) [20]. A secondary analysis of the EINSTEIN treatment program for VTE produced similar results (adjusted HR: 0.76, 95% CI: 0.46–1.25) [22]. These results were also confirmed in other studies: in a large study of 25,681 elderly patients in Canada with a history of VTE (rates: 1.55 vs. 3.47 per 100 per year, respectively; HR: 0.74; 95% CI: 0. 61–0.89) [17]; in a retrospective case–control study involving 27,862 patients in Denmark (adjusted HR: 0.72, 95% CI: 0.59–0.88) [18]; and in a prospective cohort study of 44,330 patients with a hospital diagnosis of VTE (adjusted HR: 0.74, 95% CI: 0.68 to 0.80). On the other hand, a multivariable analysis of 432 patients after discontinuation of anticoagulant therapy (HR: 1.02, 95% confidence interval: 0.36–2.91) [23] did not support this association. Similarly, in a sub-analysis of the RIETE registry (HR: 0.98, 95% CI: 0.61–1.57), no variation in VTE recurrence rates was detected between statin users and non-users [24]. Finally, a systematic review and meta-analysis [12] concluded that the use of statins is capable of reducing the risk of VTE recurrence. This meta-analysis showed a risk reduction not only during anticoagulation periods but also after discontinuation of anticoagulant therapy, indicating that statins may (probably) be able to serve as long-term pharmacological prophylactic alternatives for recurrent VTE.

### 2.3. The Risk of Bleeding Related to the Use of Statins during Anticoagulation

There are conflicting data on the impact of statins on bleeding during anticoagulation for VTE. The rate of major bleeding in statin users during anticoagulation for VTE was found to be non-significantly lower in a post hoc analysis of the EINSTEIN program for DVT and PE (adjusted HR: 0.77, 95 percent CI: 0.46–1.29). When combined with dabigatran, simvastatin and lovastatin appeared to increase the rate of bleeding (adjusted OR: 1.46, 95 percent CI: 1.17 to 1.82) [25,26]. The risk of hemorrhagic stroke was found to be 66 percent (HR: 1.68) higher in individuals taking atorvastatin, according to a post hoc analysis of the Stroke Prevention by Aggressive Reduction in Cholesterol Levels trial.

According to the Heart Protection Study, the rates of hemorrhagic strokes in statin users versus non-statin users were comparable [24]. The JUPITER study, an intervention trial evaluating rosuvastatin, suggested no differences in the incidence of intracranial hemorrhage between patients receiving 20 mg of rosuvastatin per day and those receiving placebo [13,27]. In randomized studies, the pooled risk ratio of bleeding during statin treatment was 1.10 (95 percent CI: 0.86–1.41), while in cohort studies it was 0.94 (95 percent CI: 0.81–1.10). Statins are not linked to a higher risk of intracerebral hemorrhage, according to a meta-analysis of observational studies and randomized trials [28]. The risk of bleeding and intracranial hemorrhages (ICHs) is not linked to statin therapy, according to a recent meta-analysis on the risk of bleeding that included 29 trials with 145,929 participants. However, patients who have had a stroke may be more at risk of an ICH when taking statins. Low serum cholesterol levels have been linked to an increased risk of ICH, according to epidemiological studies [29,30,31]. Potential risk factors, such as alcohol use and malnutrition, could complicate this association [32]. Statin therapy may raise the incidence of ICH, according to the SPARCL trial, a study on the use of statins in patients with prior stroke or transient ischemic attack [33]. Previous meta-analyses on the risk of ICH associated with statin treatment showed inconsistent results [34,35]. After analyzing 23 RCTs and 19 observational studies, Hackam and colleagues concluded that there was no evidence linking statins to an increased risk of ICH in either RCTs or observational studies [28]. On the other hand, recent meta-analyses have revealed that statin therapy raised the risk of ICH, particularly in patients who took high-dose statins and had a history of stroke [36,37]. The increased risk of ICH reported in the above-mentioned studies is still controversial, despite several hypotheses having been made. First, there was a suspicion that decreasing cholesterol could damage the brain’s blood vessels, leading to ICH. Furthermore, studies on animals suggested that de novo synthesis is responsible for 95% of the cholesterol present in the brain, despite the fact that the brain’s cholesterol is not influenced by variations in blood cholesterol levels [26,38]. According to a post hoc analysis of the SPARCL trial, atorvastatin-treated patients’ risk of ICH did not correlate with LDL-C levels. Instead, male sex, a history of hemorrhagic stroke, and older age account for 86% of the increased risk of ICH. The SPARCL trial did not reveal any statistically significant interaction between the use of antithrombotic drugs and statin treatment. Moreover, the THRombolysis and STatins (THRaST) trial demonstrated that the risk of hemorrhagic transformation was not increased by statin use following thrombolysis, and the risk of neurological deterioration was decreased [39]. A retrospective analysis also highlighted a reduced risk of ICH in longer-term statin users [40].

However, the available literature presents some gaps. First, most published studies do not provide a predetermined definition of bleeding or information on the likelihood of significant bleeding events. Second, few studies have examined bleeding at other sites, such as the gastrointestinal tract, which carries a significant risk of bleeding, in addition to ICH. To investigate the risk of gastrointestinal bleeding associated with the concomitant use of antithrombotic drugs and statins, further research is needed. Third, several drugs can interfere with the metabolism and absorption of statins, and the studies conducted thus far were unable to investigate drug interactions and bleeding risk. In summary, current evidence does not appear to support a correlation between statin therapy and bleeding risk. However, patients with a history of stroke seems to have a higher risk of intracranial hemorrhage, which requires further research.

### 2.4. Statin Use and Their Impact on Death during Anticoagulation for VTE

There is conflicting evidence regarding the impact of statins on mortality during anticoagulation for VTE [19,23,24]. Their ability to influence survival in patients with cardiovascular disease has been widely documented [41,42,43,44,45,46]. Several studies have shown that statins can reduce mortality during anticoagulation [41,42,43,44,45,46]. It was reported that the risk of death was halved for statin users in a Dutch registry compared to non-users (adjusted HR: 0.53, 95% CI: 0.41–0.69) [14]. A case–control study also confirmed a reduced risk of death for statin users compared to non-users (HR: 0.77, 95% confidence interval (CI): 0.71–0.84) [23]. Data from the RIETE registry, based on clinical and instrumental diagnosis of VTE including 32,062 patients with a first episode of VTE (DVT or PE), showed that patients who used statins at the time of VTE diagnosis had a significantly lower risk of death (hazard ratio (HR): 0.62, 95% confidence interval (CI): 0.48–0.79) during a course of anticoagulant therapy compared to those who did not use statins [24]. Another RIETE study found that in a subsequent cohort of 19,557 patients with VTE, the mortality rate among statin users was lower (HR: 0.68, 95% CI: 0.59–0.79) [47]. According to a third RIETE study on this topic that exclusively included patients with acute PE, statin users had a significantly lower risk of death within the first 30 days of treatment compared to non-users [48]. Using data from a large series of patients with isolated DVT, a subsequent study [49] found that statin users had a lower risk of death at three months and at one year from VTE diagnosis. This risk reduction was consistently observed during the course of anticoagulant therapy and after its discontinuation in patients with upper extremity DVT (19% at 3 months, 24% at 1 year), distal lower extremity DVT (52% at 3 months, 38% at 1 year), or proximal DVT in the lower limbs (31% at 3 months, 26% at 1 year). Risk reduction was consistently observed in both men and women, regardless of patient age, DVT risk factors, hospital size, or statin type.

## 3. The Molecular Mechanisms by Which Statins Influence Clotting Factors

The metabolism of statins involves cytochrome p450 (CYP) 3A4 in the case of simvastatin, atorvastatin, and lovastatin and CYP2C9 in the case of fluvastatin and rosuvastatin, whilst pravastatin is not markedly metabolized by CYP [50]. Both CYP3A4 and 2C9 are involved in warfarin metabolism, so the co-administration of statins, which utilize these CYP pathways, can increase INR and increase the risk of bleeding [51]. Some statins (atorvastatin and rosuvastatin) are better at reducing the potential drug–drug interactions that raise INRs (international normalized ratios).

In addition, statins as a class can reduce intravascular inflammation by a number of molecular mechanisms involving CRP (C-reactive protein), monocyte adhesion, and platelet hyper-reactivity. Statins have been demonstrated to enhance endothelial function, smooth muscle cell function, and the functioning of monocyte–macrophage cells in clinical scenarios. These positive effects may not be solely attributable to lipid-related processes but may also involve improvements in vasomotor function, the regulation of inflammatory responses, and the stability of arterial plaques [52]. Research involving animals and experiments has shown that statins can lower plasma cholesterol levels without impacting the migration or proliferation of smooth muscle cells [53]. Additionally, statins can reduce the production of matrix metalloproteinases and the in vitro accumulation of cholesterol in macrophages, thereby promoting plaque stability. Statins have been observed to decrease the synthesis of pro-inflammatory cytokines, C-reactive protein, and cellular adhesion molecules and the activation of monocytes into macrophages. Furthermore, statins reduce the adherence of monocytes to endothelial surfaces.

The underlying mechanisms by which statins reduce recurrent VTE remain largely unknown. However, a number of investigations have demonstrated that this effect is independent of plasma cholesterol levels [54,55,56]. Statins demonstrate a variety of pleiotropic qualities, such as antithrombotic and anti-inflammatory effects, in addition to their ability to lower serum cholesterol levels [57,58]. In a recent randomized controlled trial, rosuvastatin was shown to decrease thrombin generation in patients with VTE after anticoagulation withdrawal [59]. In a post hoc analysis of the same trial, rosuvastatin was also found to have fibrinolytic properties, measured by clot lysis times and plasmin inhibitor levels [60]. Moreover, another trial [61] found a 6.7 IU/dL reduction in FVIII:C levels with rosuvastatin use, suggesting a potential role of rosuvastatin in secondary prevention of VTE, since high FVIII:C levels are strongly associated with recurrent VTE. Participants with prior VTE were randomly allocated to a group to be treated with rosuvastatin 20 mg/day for 4 weeks or a no-intervention group [55,62,63]. Furthermore, atorvastatin (40 mg/day for three days) was found to increase the permeability and susceptibility to lysis of plasma fibrin clots in another open-label trial [64]. Rosuvastatin and atorvastatin have been demonstrated to downregulate molecules associated with the interleukin-6 pathway and inhibit the expression of plasminogen activator inhibitor-1 in murine models of venous thrombosis (VT), resulting in a decrease in neutrophil infiltration and an increase in thrombolysis [65,66]. Simvastatin-treated rabbit models of VT also showed comparable outcomes [67,68]. When considered collectively, these results suggest that statins may promote an anti-inflammatory and antithrombotic state in order to lower the risk of VTE recurrence. Furthermore, since residual thrombosis is a well-known risk factor for VTE recurrence, statin use can decrease the thrombus burden, reducing the inflammatory pathway created by residual thrombosis [69]. The use of statins (in combinations of types and dosages) can be categorized into three potency levels (i.e., low, medium, and high) according to their capability to reduce low-density lipoprotein cholesterol (LDL-C) [17]. Three studies [18,19,69] evaluated the correlation between potency levels and risk reductions of recurrent VTE. The incidence of recurrent PE was found to have an inverse dose–response relationship with potency category in Biere-Rafi and Schmidt’s studies [17,70,71]. The study by Tagalakis et al. [17], however, did not corroborate the above findings, as similar risk reductions were found between the high-potency group and the low-to-medium-potency group. Several studies have demonstrated the biological plausibility of statins’ protective function during anticoagulation. Statins can affect VII, VIII, and von Willebrand coagulation factors; tissue factor (TF) gene expression; D-dimer levels [72]; and TF gene expression [73,74,75].

The main biochemical mechanisms that explain how inhibition of HMG CoA is involved in various instances of clotting factor expression and function are reported below.

### 3.1. Tissue Factor

The integral membrane glycoprotein known as tissue factor (TF) is expressed constitutively on the surfaces of vascular adventitia, skin, and organs, but it is not normally expressed on monocytes or vascular endothelial cells [76,77]. Bloodstream exposure to TF occurs only following endothelial damage [78,79]. To support FVIIa binding and factor X activation, TF transitions from an inactive (or “encrypted”) state to an activated (or “decrypted”) state following cell lysis. Several studies have pointed to the impact of statins on TF. Simvastatin and fluvastatin can reduce the expression and activity of TF mRNA and inhibit nuclear factor κB (NF-κB) transcription [73]. Endothelial and vascular smooth muscle cells showed a decrease in TF mRNA expression during cerivastatin, atorvastatin, simvastatin, pravastatin, lovastatin, and fluvastatin therapy [76]. Fluvastatin and simvastatin also inhibit tissue factor (TF) activity in a dose-dependent manner [77]. During statin therapy, aortic smooth muscle and endothelial cells showed a decrease in TF expression and activity [78] in hypercholesterolemic patients [74]. By inhibiting ERK1/2 phosphorylation and relocating PAR1, rosuvastatin and fluvastatin can prevent the induction of TF expression. Additionally, in animal models, statins have been shown to downregulate TF [80,81,82,83].

### 3.2. Trombin

*Thrombin* is the main initiator of physiological blood coagulation and pathological thrombosis [84]. Thrombin exerts its activity as a cofactor for activated FVII (FVIIa) and, in addition, is involved in fibrinogen regulation, FV activation, endothelial cell activation, platelet activation, increased TF expression, cell proliferation, and vascular constriction and mediates thrombin functions [85,86]. Experimental and clinical data suggest that the use of statins lowers thrombin levels [87,88,89,90]. In individuals with hypercholesterolemia, simvastatin has been shown to reduce thrombin production [91,92]. In hypercholesterolemic individuals, pravastatin can decrease platelet-dependent thrombin generation [93,94]. Simvastatin prevents the production of thrombin in both healthy and hypercholesterolemic individuals [95]. Regardless of blood cholesterol levels, these subjects showed a reduction in prothrombin depletion rate of 16 percent and a reduction in prothrombin activation product formation rate of 27 percent [96]. Regardless of cholesterol reduction, simvastatin can lower thrombin generation in hypercholesterolemic patients at high cardiovascular risk [97]. In a similar way, atorvastatin reduces thrombin formation by 30–40% [98]. In hypercholesterolemic patients, simvastatin decreased the rate at which thrombin was generated in response to vascular damage [95]. Elderly atrial fibrillation patients receiving oral anticoagulant therapy have also been shown to have reduced thrombin generation [98]. Statins are useful in lowering thrombin generation even in patients with type 2 diabetes [99,100,101]. Thrombin is connected to a number of atherogenic processes, including inflammation, leukocyte trafficking, and cell migration and proliferation [55].

### 3.3. Fibrinogen

Since *fibrinogen* serves as thrombin’s primary substrate, high fibrinogen levels are associated with an increased risk of thrombosis. There are contrasting data on how statins affect fibrinogen levels. The use of simvastatin in hypercholesterolemic patients decreases fibrinopeptides independently of cholesterol’s serum reduction [88,89]. A study by Undas et al. suggests that statins can modify fibrin clot properties and enhance the permeability and lysability of fibrin clots [55].

### 3.4. Factor VII

The impact of statins on *FVIIa* activity and levels is a matter of debate. Independently of cholesterol-lowering effects, atorvastatin lowers factor VII antigen levels and FVII coagulant activity in hypercholesterolemic patients [102,103,104,105]. On the other hand, an in vitro study revealed that FVIIa activity was not modified during statin therapy [103]. In hypercholesterolemic patients, higher doses of atorvastatin decreased FVII activity by 8% [104]. It seems that simvastatin [106], pravastatin [107], and fluvastatin [108] have no effect on FVII levels. However, further studies conducted specifically on this topic are needed. Table 1 offers a summary of the principal molecular mechanisms by which statins affect coagulation.

## 4. Discussion

From some years, there has been growing interest in statins’ antithrombotic proprieties. Many studies have been performed on the beneficial effects of statins on cardiovascular risk, mainly due to their ability to impact cholesterol serum levels. Anecdotal evidence of the influence of statins on coagulation was obtained as early as the 1990s and 2000s. According to preliminary data, fluvastatin and simvastatin may both lower the expression of the tissue factor (TF) gene [73,74]. Independently of the reduction in blood cholesterol, the anticoagulant effects of statins are greater in subjects with hypercholesterolemia. Statins inhibit blood clotting by downregulating tissue factor expression and upregulating endothelial tissue mediator expression [72,73,74,75,76,77,78]. This leads to a decrease in thrombin production and, in turn, to a decrease in the number of procoagulant reactions that thrombin catalyzes. Statins’ ability to lower VTE recurrences and death during anticoagulation for VTE may be partially explained by their anticoagulant properties. It is unclear if the anticoagulant properties of different types of statins are supported by similar mechanisms [47]. Several underlying mechanisms have been suggested. Virchow’s triad, which includes hypercoagulability, venous stasis, and endothelial injury/dysfunction, is the primary risk factor for venous thrombosis. The production of thrombin [58] and other coagulation factors can be inhibited by statins. Therefore, the antithrombotic characteristic of statins may have an impact on preventing VTE. Second, VTE is linked to the activation of several inflammatory markers, including IL-6, IL-8, C-reactive protein, and tumor necrosis factor. Thus, it is possible that statins’ anti-inflammatory properties contribute to the prevention of VTE. The primary impact of statins is widely recognized to be the lowering of blood cholesterol levels, but other pleiotropic effects can explain their biological activities in VTE [54].

Some considerations can be made:

Statins seem not to have an effect on recurrence rates in patients with an unprovoked first event of VTE. Although rosuvastatin [13] (one of the statins most studied in relation to this topic) significantly reduced the incidence of symptomatic VTE in a randomized trial in apparently healthy individuals, there is uncertainty regarding the recommendability of rosuvastatin and statins in general for primary VET prevention [13]; the question is still a matter of debate, and their efficacy needs to be confirmed by more high-quality studies, especially ad hoc randomized clinical trials.

Statin therapy can reduce the risk of recurrent VTE and mortality, in addition to all-cause mortality in patients with high cardiovascular risk factors [16,17,18,19,20].

The beneficial effect of statins may be higher in high-risk VTE patients, especially those with high cardiovascular risk factors, but this benefit appears not to be so strong in low- or moderate-VTE-risk patients.

Statins seems to have a potential role in secondary prevention of VTE, but their use as an associated therapy (with canonical anticoagulation therapy) during long-term treatment of VTE needs to be confirmed by future studies, such as randomized trials.

Rosuvastatin 20 mg daily is the most-used type of statin in RCT trials, while in most cohort studies [13], the statin type ranges from simvastatin to lovastatin, though there are few studies that have compared statin types and dosages. In a study performed on the RIETE registry, the authors compared different types [47] of statins and the all-cause mortality of patients with VTE during anticoagulation. Compared to non-statin users, patients on simvastatin or other statins had a lower mortality rate, but there was no significant difference in mortality among patients on atorvastatin or rosuvastatin. Similar data were suggested in a study in which the strength of associations between stratified intensities of statin therapy and mortality was assessed [47]. It was observed that although low and medium doses of statins decreased the prevalence of fatal PE, there was no significant difference at high doses.

Statins have a protective role against short-term mortality in acute symptomatic PE [48].

Statins have a protective role against mortality in patients with deep vein thrombosis [49].

The underlying mechanisms by which statins reduce recurrent VTE remain largely unknown. However, a number of investigations have demonstrated that this effect is independent of plasma cholesterol levels and that it is mediated by statins’ effect on blood clotting [54].

Successful prevention in patients could have a major clinical impact, considering the high morbidity and mortality in these patients. Although there is an open debate on the appropriateness of statin treatment for the prevention of VTE, their usefulness seems to be real, and several underlying mechanisms have been proposed.

First, venous stasis, hypercoagulability, and endothelial injury/dysfunction (known as Virchow’s triad) are the most important risk factors for venous thrombosis [54]. Statins have been shown to prevent the generation of thrombin and other coagulation factors in basic and clinical studies. Thus, the effect of statins on the prevention of VTE may be related to their antithrombotic properties. Second, VTE is related to the activation of inflammatory markers, such as C-reactive protein, IL-6, IL-8, and tumor necrosis factor, and it has generally been considered as an inflammatory disease of the blood vessels [54]. It has been reported that their anti-inflammatory properties are some of the pleiotropic effects of statins [54]. The anti-inflammatory effect of statins may also play a role in the prevention of VTE. Third, it is well known that the main effect of statins is to reduce blood lipid levels. Several studies found that elevated low-density lipoprotein cholesterol (LDL-C) was significantly associated with increased risk of VTE [54]. Therefore, the efficacy of statins in lowering lipid levels may contribute to their prevention of VTE.

At this time, there is not sufficient evidence to recommend routine statin treatment in the primary care of populations for VTE prevention. However, in patients with a high risk of VTE (e.g., cancer), statins could be considered as an adjunct therapy for preventing incident VTE. Statins could be prescribed for patients with prior VTE to prevent recurrence, without increasing the risk of bleeding.

## 5. Conclusions

**Figure 1:**
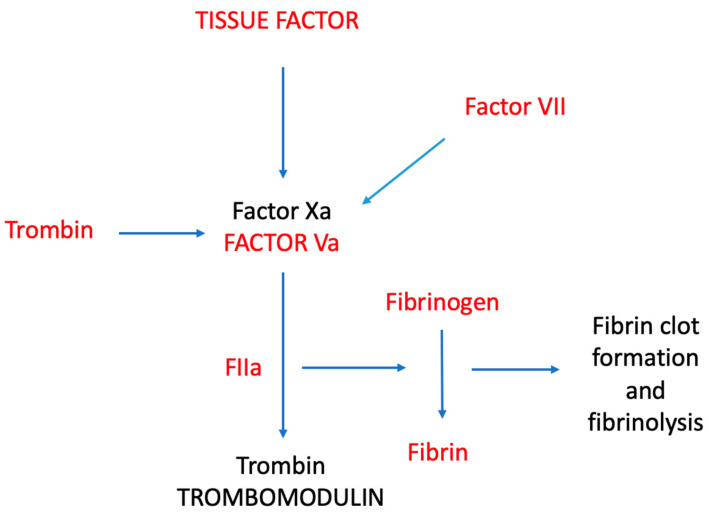
Influence of statins on different clotting factors. The main clotting factors inhibited by statins are shown in red.

In conclusion, several studies have shown that statins possess an anticoagulant effect through their ability to modulate tissue factor (TF) expression, thrombin generation, and most of the thrombin-catalyzed pro-coagulant reactions (i.e., those involving fibrinogen and the activation of factor V and factor XIII). Increased fibrinolytic activity and endothelial thrombomodulin expression both contribute to the anticoagulant effects of statins. The anticoagulant effects of statins are also mediated by increased expression of endothelial thrombomodulin and increased fibrinolytic activity. In addition to these direct antithrombotic activities, additional plausible properties of statins (i.e., anti-inflammatory and/or antioxidant activities) may indirectly interfere with thrombus formation and propagation. Some research suggests that the ability of statins to lower primary and secondary VTE as well as death may be due to their anticoagulant-induced effects. Nevertheless, there is ongoing discussion regarding the effect of statins on VTE, and the data regarding their efficacy in preventing venous thromboembolism are inconsistent. Their presumed protective role against VTE is a matter of debate, and they should not be considered a substitute for guideline-recommended VTE prophylaxis or anticoagulation treatment in VTE patients [104]. Further studies specifically performed on this topic are needed. Figure 1 summarizes the principal activities of statins with respect to clotting factors.

## Figures and Tables

**Table 1:** Molecular mechanisms by which statins affect coagulation.

Substrate	Physio-Pathological Pathway	Proposed Effect of Statin	Molecular Mechanisms	References in the Main Text
Tissue factor	Statins are expressed constitutively on the surfaces of vascular adventitia, skin, and organs but are not normally expressed on monocytes or vascular endothelial cells. Upon binding and factor X activation, TF transitions from an inactive (or “encrypted”) state to an activated (or “decrypted”) state following cell lysis	Experimental and clinical data suggest that the use of statins lowers thrombin levels	Reduce expression and activity of TF mRNA; inhibit nuclear factor κB (NF-κB) transcription; decrease TF mRNA expression; inhibit tissue factor (TF) activity in a dose-dependent manner; inhibit ERK1/2 phosphorylation and PAR1 relocation	[73,74,75,76,77,78,79]
Thrombin	Statins are the main initiators of physiological blood coagulation and pathological thrombosis and exert their activity as cofactors for activated FVII	Statins lower thrombin levels	Thrombin exerts its activity as a cofactor for activated FVII (FVIIa) and, in addition, is involved in fibrinogen regulation, FV activation, endothelial cell activation, platelet activation, increased TF expression, cell proliferation, and vascular constriction and mediates thrombin functions. Statins lower thrombin levels and affect this pathway	[80,81,82,83,84,85,86,87,88,89,90,91,92,93,94,95,96,97,98]
Fibrinogen	*Fibrinogen* serves as thrombin’s primary substrate; high fibrinogen levels are associated with an increased risk of thrombosis	Statins lower fibrinogen levels	The use of simvastatin in hypercholesterolemic patients decreases fibrinopeptides independently of cholesterol’s serum reduction. Statins can modify fibrin clot properties and enhance the permeability and lysability of fibrin clots	[88,89,90,91,92,93,94,95,96,97,98,99]
FVIIa	One of the major coagulation factors implicated in the coagulation cascade	Statins seem to reduce FVIIa activities; however, there are still some doubts about their role	In hypercholesterolemic patients, higher doses of atorvastatin decrease FVII activity by 8%. It seems that fluvastatin, pravastatin, and simvastatin have no effect on FVII levels	[55,100,101,102]
